# The interactive effect of preoperative consultation and operating room admission by a counselor on anxiety level and vital signs in patients undergoing Coronary Artery Bypass Grafting surgery. A clinical trial study

**DOI:** 10.17533/udea.iee.v38n2e07

**Published:** 2020-07-10

**Authors:** Zandi Shirdel, Imani Behzad, Babak Manafi, Mehdi Saheb

**Affiliations:** 1 M.Sc. Student, Department of Operating Room, Student Research Committee, Hamadan University of Medical Sciences, Hamadan, Iran. Email: shirdel.zandi1994@yahoo.com Hamadan University of Medical Sciences Iran shirdel.zandi1994@yahoo.com; 2 Assistant professor, Department of Operating Room, School of Paramedicine, Hamadan University of Medical Sciences, Hamadan, Iran. Email: behzadiman@yahoo.com. Corresponding author. Hamadan University of Medical Sciences Iran behzadiman@yahoo.com; 3 Assistant professor, Department of heart surgery, School of medicine, Hamadan University of Medical Sciences, Hamadan, Iran. Email: manafi@umsha.ac.ir Hamadan University of Medical Sciences Iran manafi@umsha.ac.ir; 4 BSc. Operating Room. Farshchian Hospital, Hamadan University of Medical Sciences, Hamadan, Iran. Email: Mahdi.Sahebi1415@yahoo.com Hamadan University of Medical Sciences Iran Mahdi.Sahebi1415@yahoo.com

**Keywords:** coronary artery bypass, respiratory rate, blood pressure, temperature, heart rate, operating rooms, counselors, anxiety, control groups., puente de arteria coronaria, frecuencia respiratoria, presión sanguínea, temperatura, frecuencia cardíaca, quirófanos, consejeros, ansiedad, grupos control., ponte de artéria coronária, taxa respiratória, pressão sanguínea, temperatura, frequência cardíaca, salas cirúrgicas, conselheiros, ansiedade, grupos controle.

## Abstract

**Objective.:**

The purpose of this study was to provide appropriate preoperative supportive conditions to improve anxiety and vital signs for patients undergoing Coronary Artery Bypass Grafting -CABG- surgery.

**Methods.:**

This clinical trial study was performed on 90 patients undergoing CABG surgery in Farshchian Hospital of Hamadan, Iran in 2019. Sample was selected by convenience and were randomly divided into three groups: control (*n*=30), intervention1 (*n*=30), and intervention2 (*n*=30). The control group received only the routine preoperative counseling of ward and admitted to the operating room as usual; the intervention1 and intervention2 groups in addition received another two counseling sessions, then the intervention1 group was admitted in the operating room as usual, but the intervention2 group was admitted by the counselor in the operating room. Data were collected using a three-part questionnaire including demographic characteristics, vital signs chart, and the Spielberger's State-Trait Anxiety Inventory.

**Results.:**

The results showed that there was a significant difference in the mean anxiety of the three groups after admission in the operating room (intervention2 was lower than intervention1 and control groups, *p*<0.001; and intervention 1 group was lower than control group, *p*<0.001) and also there was a significant difference between the mean systolic blood pressure, heart rate and respiratory rate of the three groups (*p*<0.001) but the mean of the variables of temperature and diastolic blood pressure in the three groups were not significantly different (*p*=0.59 and *p=*0.225, respectively).

**Conclusion.:**

Our results revealed preoperative consultation and admission in the operating room by the consultant can reduce the level of anxiety and stability of vital signs of patients undergoing CABG.

## Resumen

**Objetivo.:**

Evaluar si las condiciones de apoyo preoperatorias mejoran la ansiedad y los signos vitales para los pacientes sometidos a cirugía de bypass de arterias coronarias (CABG por sus siglas en inglés).

**Métodos.:**

Este estudio clínico se realizó en 90 pacientes sometidos a cirugía de revascularización coronaria -CABG- en el Hospital Farshchian de Hamadan en Irán. La muestra se seleccionó por conveniencia y se dividió aleatoriamente en tres grupos: control (*n*=30), intervención1 (*n*=30) e intervención2 (*n*=30). El grupo de control recibió solamente la consejería preoperatoria de rutina cuando ingresó a hospitalización y en el momento de admisión en la sala de cirugía; los grupos de intervención1 e intervención2 además de la consejería rutina recibieron otra sesión adicional; y cuando se admitió el grupo de intervención2 en la sala de operaciones, lo acompañó la misma persona que hizo la consejería. Los datos se recopilaron mediante un cuestionario de tres partes que incluyó características demográficas, un cuadro de signos vitales y los puntajes del *State-Trait Anxiety Inventory de Spielberger*.

**Resultados.:**

Los hallazgos mostraron que hubo diferencia significativa en el puntaje de ansiedad de los tres grupos después de la admisión en la sala cirugía (en intervención2 fue menor que en los grupos intervención1 y control, *p*<0.001; y en el grupo de intervención1 fue menor que en el grupo de control, *p*<0.001). También se encontraron diferencias estadísticamente significantes para las diferencias en la presión arterial sistólica, la frecuencia cardíaca y la frecuencia respiratoria de los tres grupos, pero no para las variables de temperatura y presión arterial diastólica (*p*=0.59 y *p*=0.225, respectivamente).

**Conclusión.:**

Nuestros resultados revelaron que la consulta preoperatoria y la admisión en el quirófano por parte del consultor pueden reducir el nivel de ansiedad y la estabilidad de los signos vitales de los pacientes sometidos a CABG.

## Resumo

**Objetivo.:**

O propósito deste estudo foi avaliar se as condições de apoio pré-operatórias melhoram a ansiedade e os signos vitais para os pacientes submetidos a cirurgia de bypass de artérias coronárias (CABG por suas siglas em inglês).

**Métodos.:**

Este estudo clínico se realizou em 90 pacientes submetidos a cirurgia de revascularização coronária -CABG- no Hospital Farshchian de Hamadan no Irão. A mostra se selecionou por de conveniência e se dividiu aleatoriamente em três grupos: controle (*n*=30), intervenção1 (*n*=30) e intervenção2 (*n*=30). O grupo de controle recebeu somente o aconselhamento pré-operatória de rotina quando ingressou a hospitalização e no momento de admissão na sala de cirurgia; os grupos de intervenção1 e intervenção2 ademais do aconselhamento de rotina receberam outra sessão adicional; e quando o grupo de intervenção2 foi admitido na sala de operações foi acompanhado pela mesma pessoa que fez o aconselhamento. Os dados se recopilaram mediante um questionário de três partes que incluiu características demográficas, um quadro de signos vitais e as pontuações do State-Trait Anxiety Inventory de Spielberger.

**Resultados.:**

As descobertas mostraram que houve diferença significativa na pontuação de ansiedade dos três grupos depois da admissão na sala cirurgia (em intervenção2 foi menor do que nos grupos intervenção1 e controle, *p*<0.001; e no grupo de intervenção1 foi menor do que no grupo de controle, *p*<0.001). Também se encontrou diferenças estatisticamente significantes para as diferenças na pressão arterial sistólica, a frequência cardíaca e a frequência respiratória dos três grupos, mas não para as variáveis de temperatura e pressão arterial diastólica (*p*=0.59 e *p*=0.225, respectivamente).

**Conclusão:**

Nossos resultados revelaram que a consulta pré-operatória e a admissão na sala de cirurgia por parte do consultor podem reduzir o nível de ansiedade e a estabilidade dos signos vitais dos pacientes submetidos a CABG.

## Introduction

Cardiovascular disease has become a major worldwide problem and is a serious health threat that causes numerous biological, psychological and social problems and is one of the leading causes of death and disability.(1) These diseases also impose a huge cost on the health system of countries.(2) According to the World Health Organization, 17.7 million people died of cardiovascular diseases in 2016, and cardiovascular diseases account for 31% of all global deaths.(3) According to the Global Borden Disease Study Center in Iran, the rate of death from cardiovascular disease has increased from 31.9% in 1990 to 46.8% in a decade,(4) and represent 43% of the total number of deaths.(3) Coronary Artery Bypass Graft (CABG) surgery is one of the most commonly used surgical procedures for patients with coronary artery stenosis.(5) Annually, 30 000 to 40 000 people undergo cardiac surgery, and CABG constitutes 50-60% of these surgeries.(6) One of the frequent problems of patients before the surgery is higher levels of anxiety in this case as compared to that in other surgeries, since heart is closely related to human life and death.(7) 

Actually Cardiac surgery is often associated with anxiety and fear in patients undergoing it.(8) Anxiety before cardiac surgery can lead to stimulates the sympathetic system and significant changes in the patient's heart rate and blood pressure,(9,10) elevates the risk of mortality,(11) increases the intraoperative anesthetic requirement and can prolonged recovery.(12) ways to control anxiety include medication and non-medication.(6) Routine use of anxiolytic medications to reduce anxiety in patients with pain can prevent the reporting of pain due to drowsiness, pulmonary complications due to reduce her ability to breathe deeply, get out of bed, or participate in treatment.(7,13) Non-pharmacological approaches such as inhalation aromatherapy,(14) massage therapy,(15) and music therapy,(16) Visitation by Operating Room Staff,(17) and preoperative education programs(18) can help reduce anxiety. Among the measures the nurse can take to minimize anxiety before cardiac surgery, giving information about surgery, promoting acquaintance dialogue, and understanding the patient are more important strategies.(19)

Given that the receptionist in the operating room is one of the last people the patient will be in contact with the patient before surgery, The researcher speculates that if this is a relationship with the patient who was already in touch with the patient, a positive interaction and Having a friendship between them will affect the patient's anxiety and vital signs. According to the researcher, no study has been conducted in this area, so the present study will be conducted to determine the interactive effect of preoperative consultation and patient admission in the operating room by counselor on the level of anxiety and vital signs in coronary artery bypass graft surgery (CABG).

## Methods

The present study was a clinical trial study performed at Farshchian Hospital of Hamadan, Iran in April 2019. Using convenience sampling, 90 patients were selected for CABG surgery and were randomly divided into three groups (control, intervention 1 and intervention 2) using random sampling and weekly block method. The inclusion criteria were include the at least 18 years of age; fully conscious and knowledgeable regarding the time, place, and person; having the ability to communicate, not having a history of heart surgery, lacking physical and cognitive disorders; lacking medical education or what was related to it; not having a drug addiction; no prior medical diagnosis of anxiety and depression and not taking tranquilizer, anti-depressants, or anti-anxiety drugs at one month before surgery. The exclusion criteria were the unwillingness of patients to continue the participation in the study and their condition worsening during the study period.

The data collection tool consisted of three parts: personal traits, medical data, and Spielberg state anxiety Inventory. The state of anxiety is the same instantaneous individual anxiety expressing the person’s current feeling or emotion at a time period like getting prepared for the surgery. The Spielberg anxiety questionnaire had global validity and reliability. The validity of this test, in order to reach a meaningful result, the mean anxiety for the standard and normal community was compared at 1% and 5% across all age groups, indicating the validity of the measurement of anxiety. The reliability of the Spielberger's State-Trait Anxiety Inventory has been calculated to be 0.90 and 0.86 in the studies by Roohy *et al.* (20) and Safara *et al.,* (21) respectively. In addition, its reliability and validity in the Iranian society cardiac patients was confirmed via the study of Akbarzade *et al*.(22) The questionnaire was made up of 20 multiple choice questions, with the options of “very little, little, a lot, and very much”. The minimum score for this questionnaire was 20 and the maximum was 80.

After consenting to the samples at the first visit and before any intervention, the patient completed a three-part questionnaire as a pre-test. The control group received only the routine counseling program that the ward provides for all patients, but intervention 1 and intervention 2 group’s in accordance with Table 1 received two one-hour counseling sessions in the day before surgery in addition to the routine counseling program. Counseling sessions were organized individually for each patient by the counselor, counseling sessions were held in the conference room of the ward, and the counselor, the patient and the experienced nurse were present at these sessions. At admission to the operating room, the control group and the intervention group 1 samples were admitted to the operating room according to the routine program of the hospital with a non-consultant in the operating room. But in order to admission Intervention Group 2 patients in operating room, they were accompanied by a consultant and patient transfer team from the ward to the operating room; consultant would admitted the patient in the operating room and perform the tasks performed on the patient's admission in the operating room; while waiting for anesthesia, the consultant stayed with the patient and acted as an emotional support and mentor in the operating room for the patient by benefiting from the positive interaction that was created during the two meetings with the patient. Then before receiving anesthesia, their vital signs and state anxiety were recorded as a post-test. 

At the beginning of the counseling program and after introducing herself to the patients and explaining the purpose of the study, the researcher obtained consent from all patients. Also, participants were assured that all information collected from them would remain confidential. Finally, the data resulting from descriptive statistics, independent T-test, analysis of variance, analysis of covariance and ANOVA at the significant level of *p*<0.05. Were analyzed by SPSS V.23.

**Table 1:** Grouping and scheduling of consultation sessions and interventions

Session	Group	Meeting time	Topics discussed
First session	Intervention1 & Intervention2	The day before surgery at 10 am	Introduce the consultant to the patient - Information about the nature of the disease - Type of disease - Cause of the disease - Cause of surgical diagnosis - Surgical technique - Surgical complications - Anesthesia information - Anesthesia complications
Second session	Intervention1 & Intervention2	The day before surgery at 6 pm	Answering questions about the first session - Providing information about the post-surgical course - Getting to know the patient with ICU - Providing information about self-care at home.

## Results

The results of this study showed that the mean age of the control, intervention 1 and intervention 2 groups were 61.9±8.02 years, 62.13±7.04 years, and 65.6±9.3 years, respectively and Mean BMI of the control, intervention 1 and intervention 2 groups were 24±3.2, 25.6±2.4 and 25.6±4 respectively In addition, no significant difference was observed in the age and BMI by the ANOVA test. Most of the patients in these three groups were male (68.9%) and most of the subjects were illiterate (55.6%) and the results of Chi-square test showed no significant difference between the three groups in terms of gender and education (Table 2).

**Table 2:** Demographic characteristics of the group

Variable	Variable	Control	Intervention1	Intervention2	Total	***p*-value**
Age	Age	61.9±8.0	62.1±7.0	65.6±9.3	63.2±8.3	0.15
BMI	BMI	24±3.2	25.6±2.4	25.6±3.9	25.1±3.3	0.08
Sex	Male	21 (70%)	20 (66.7%)	21 (70%)	62 (68.9%)	0.9
Sex	Female	9 (30%)	10 (33.3%)	9 (30%)	28 (31.9%)	
Education	Illiterate	17 (56.7%)	16 (53.3%)	17(56.7%)	50 (55.6%)	0.6
Education	Under the diploma	10 (33.3%)	10 (33.3%)	8 (26.7%)	28 (31.1%)	
Education	Diploma	3 (10%)	4 (13.3%)	3 (10%)	10 (11.1%)	
Education	Academic	0	0	2 (6.7%)	2 (2.2%)	

One-way covariance analysis test was used to compare the pre-test score of anxiety variables and vital signs. According to the results, the *p*-values in the pre-test of the variables of heart rate, respiratory rate, systolic blood pressure and positional anxiety showed no significant difference between the three groups. But for the variables of temperature and diastolic blood pressure were significant at the 0.05 level (Table 3).

**Table 3:** Covariance analysis of Pre-test vital signs and situational anxiety

Variable	Variable	Sum of squares	DF	Mean squares	F	***p*-value**
Heart rate	Between groups	197.1	2	98.5	2.616	0.079
Heart rate	Within group	3276.5	87	37.6		
Heart rate	Total	3473.6	89			
Respiratory rate	Between groups	17.1	2	8.5	2.556	0.083
Respiratory rate	Within group	291.9	87	3.3		
Respiratory rate	Total	309.1	89			
Temperature	Between groups	1.8	2	0.9	6.836	0.002
Temperature	Within group	11.5	87	0.1		
Temperature	Total	13.4	89			
Systolic blood pressure	Between groups	774.6	2	387.3	2.368	0.100
Systolic blood pressure	Within group	14229.4	87	163.5		
Systolic blood pressure	Total	15004.0	89			
Diastolic blood pressure	Between groups	898.5	2	449.3	5.776	0.004
Diastolic blood pressure	Within group	6769.0	87	77.8		
Diastolic blood pressure	Total	7667.8	89			
Anxiety	Between groups	66.6	2	33.3	0.607	0.547
Anxiety	Within group	4771.9	87	54.8		
Anxiety	Total	4838.5	8			

Covariance analysis test revealed that collaboration effect of preoperative counseling and patient admission in operating room by counselor on anxiety, heart rate, respiratory rate and systolic blood pressure has been meaningful, but it was not significant on temperature and diastolic blood pressure (Table 4).

**Table 4:** Covariance analysis of Post-test vital signs and situational anxiety

Variable	Variable	Sum of squares	DF	Mean squares	F	***p*-value**
Anxiety	Between groups	4525.3	2	2262.7	109.22	0.001
Anxiety	Within group	1719.4	83	20.7		
Anxiety	Total	225396.0	87			
Heart rate	Between groups	973.5	2	486.7	19.93	0.001
Heart rate	Within group	2100.0	87	25.4		
Heart rate	Total	609698.0	89			
Respiratory rate	Between groups	292.0	2	146.0	135.43	0.001
Respiratory rate	Within group	92.7	86	1.1		
Respiratory rate	Total	29937.0	90			
Temperature	Between groups	1.1	2	0.5	0.51	0.59
Temperature	Within group	99.2	86	1.2		
Temperature	Total	123920.1	90			
Systolic blood pressure	Between groups	1383.5	2	691.8	12.74	0.001
Systolic blood pressure	Within group	4668.1	86	54.3		
Systolic blood pressure	Total	1351381.0	90			
Diastolic blood pressure	Between groups	38.8	2	19.4	1.52	0.225
Diastolic blood pressure	Within group	1100.4	86	19.4		
Diastolic blood pressure	Total	482400.0	90			

In the following, Tukey's post hoc test was used to compare two-way mean anxiety, respiratory rate and systolic blood pressure in the control and intervention groups. Based on the results, there was a significant difference in the level of anxiety between the control group and the intervention group 1 and 2 (*p*<0.001). There was also a significant difference in the level of anxiety between intervention group 1 and intervention 2 (*p*<0.001). accordingly, the mean of anxiety in intervention 2 was lower than the control group and intervention 1 and the mean of anxiety in intervention 1 group was lower than control group (Table 5).between the mean of heart rate in the control group with intervention 1 (*p*=0.004), between the mean of heart rate in the control group with intervention 2 (*p*=0.001) and between the mean of heart rate in the intervention 1 group with intervention 2 (*p*=0.002) there was a significant difference that mean of heart rate in intervention 2 was lower than the control group and intervention 1 and the mean of heart rate in intervention 1 group was lower than control group (Table 5).

 Between the mean of respiratory rate in the control group with intervention 1 and 2 groups (*p*<0.001) and also between the mean of respiratory rate in the intervention group 1 and intervention 2 group (*p*<0.001) there was a significant difference. It’s that the mean of respiratory rate in intervention 2 was lower than the control group and intervention 1. The mean of respiratory rate in intervention 1 group was lower than control group (Table 5). Between the mean of systolic blood pressure in the control group with intervention 1 (*p*=0.018), between the mean of systolic blood pressure in the control group with intervention 2 (*p*<0.001) and between the mean of systolic blood pressure in the intervention 1 group with intervention 2 (*p*=0.008) there was a significant difference that the mean of systolic blood pressure in intervention 2 was lower than the control group and intervention 1 and the mean of systolic blood pressure in intervention 1 group was lower than control group (Table 5).

**Table 5:** Pairwise comparisons

Variable	Type of comparison	Mean difference	Std. error	***p*-value**
Anxiety	Control - intervention1	8.0	1.0	<0.001
Anxiety	Control - intervention2	17.6	1.2	<0.001
Anxiety	Intervention1- intervention2	9.6	193.1	<0.001
Heart rate	Control - intervention1	3.8	1.3	0.004
Heart rate	Control - intervention2	8.1	1.2	<0.001
Heart rate	Intervention1 - intervention2	4.2	1.3	0.002
Respiratory rate	Control - intervention1	1.2	0.3	<0.001
Respiratory rate	Control - intervention2	4.3	0.3	<0.001
Respiratory rate	Intervention1 - intervention2	3.1	0.3	<0.001
Systolic blood pressure	Control - intervention1	4.6	1.9	0.018
Systolic blood pressure	Control - intervention2	9.8	1.9	<0.001
Systolic blood pressure	Intervention1 - intervention2	5.2	1.9	0.008

## Discussion

The results of this study show that there is no significant difference between the pre-test anxiety scores of the three groups, which is in agreement with the results of studies such as Fazlollahpour et al.,(23) Amiri *et al.*(24) and Abbasi *et al*.(25) It has also been shown that pre-test three vital signs including systolic blood pressure, heart rate, and respiratory rate were not significantly different between the three groups, which is consistent with the findings of Amiri *et al.*,(24) Abbasi *et al*.(25) and Hemmati *et al.(*26) However, there were significant differences between the two groups in the pre-test of diastolic blood pressure and temperature variables, which is inconsistent with the results of Abbasi and Hemmati's study.(25,26) The results show that there is a significant difference between the mean scores of post-test anxiety of the control group with the intervention group 1 and intervention 2, which Indicates the effectiveness of the preoperative counseling program that this consistent with the studies of Karama *et al*.,(27) Rosiek *et al.(*28) and Timurie *et al*.(6)

The results also show that there is a significant difference between the post-test scores of the two groups of intervention 1 and intervention 2, which indicates the effectiveness of consultant admission in the operating room which no relevant article has been found in relation to this finding to compare the results that this indicating the novelty and importance of this study.

This study showed that there is a significant difference between the three variables of heart rate, respiration and systolic blood pressure in the control group with the intervention group 1 and intervention group 2, which indicates the effectiveness of preoperative counseling on these variables. This is consistent with the findings of the study by Amiri *et al*.,(24) Hasan Genc *et al*.,(29) Degirmen *et al*.,(30) but is inconsistent with the results of the study by García Sierra *et al.(*31) The results also indicate that there is a significant difference in pre-test of these three variables between the two groups of intervention group 1 and intervention group 2 which indicates the effectiveness of consultant admission in the operating room on these variables no study was found to compare these results that this indicating the novelty and importance of this study. But the results of this study showed that comparison of post-test and pre-test two variables of temperature and diastolic blood pressure were not significantly different in the three groups. This finding is in line with the results of studies such as the García Sierra *et al*.(31) and is inconsistent with the findings of the Zarei *et al.*,(32) and Degirmen *et al.* (30) studies.

Based on the findings, it can be concluded that preoperative consultation and admission in operating room by counselor can reduce the level of anxiety and stability of vital signs in patients. According to the data it can be decided that the admission in operating room by counselor will increase the effectiveness of the surgical consultation, reduce the level of anxiety and stabilize the vital signs of the patients. This approach can be considered as a strength and superiority of the present study than previous studies which suggest that cardiac surgery centers should adopt these results in order to reduce the anxiety and stability of the vital symptoms of patients.
